# Case report of LCP pediatric hip osteosynthesis of a proximal femoral fracture in a child with marble bone disease

**DOI:** 10.11604/pamj.2013.15.66.2637

**Published:** 2013-06-23

**Authors:** Hristo Georgiev, Venelin A Alexiev

**Affiliations:** 1 Department of Paediatric Orthopaedics, Sofia Medical University, Bulgaria

**Keywords:** Osteopetrosis, pathologic intertrochanteric fracture, child, open reduction, LCP Paediatric Hip

## Abstract

We present a case report demonstrating the experience of the department of pediatric orthopaedics of the University Orthopedic Hospital at Sofia Medical University in the treatment of an intertrochanteric proximal femoral fracture in a child with osteopetrosis. We performed open fracture reduction with stable 120° LCP (Locking Compression Plate) Pediatric hip osteosynthesis. Fracture consolidation and ability to walk without crutches was achieved in a half a year. The presented case is the first for Bulgaria. There are still no publications in the world literature on application of such osteosynthesis in marble bone disease.

## Introduction

Osteopetrosis (marble bone disease) [1] is a group of extremely rare bone dysplasias. The pathology is chracterized by increased bone density due to osteoclastic dysfunction. The osteoclasts do not resorb bone tissue and enchondral cartilage thus leading to impaired bone remodelling and accumulation of large quantities of immature bone. The most common fractures in marble bone disease are in the proximal femur. In the severe (early onset) with autosomal-recessive inheritance form, the pathologic fractures occur in early infancy. The presentation of the milder (late onset, osteopetrosis tarda) form with autosomal-dominant inheritance, varies from asymptomatic course, detected incidentally on X-ray, to occurrence of frequent fractures of the long and short bones — up to 78% of the patients [2]. Usually their conservative treatment is unsuccessful, while the operative treatment is technically difficult and followed by complications due to defective bone marrow function, delayed consolidation and higher risk of additional fractures during surgical application of orthopedic ostheosynthesis.

## Patient and observation

Our clinical case is a 13 year old girl, born from a second delivery with pathologic contractions in 5 l.m. and maternal edema with albuminuria at the end of the pregnancy. Delivery was normal; with birth weight 3 500 grams and 50 cm in length fetus. In the neonatal period were detected dolichocephalism, hepatosplenomegaly, amaurosis, osteosclerotic bone changes on X-ray, hematologic aberrations; the iagnosis of osteopetrosis was made. Corticosteroid therapy was instituted. In November 2008 the patient suffered from edema in the right mandible and she was surgically treated for osteomyelitis. After 2009 there is a lack of genetic and hematologic control. The child attends a special school for blind with very high success. She is independent in everyday living, intellectually she is absolutely intact.

In February 2012, she was diagnosed with intertrochanteric fracture of her left femur after a trauma and was referred to our department. On admission, the child was in an impaired general status, non-ambulatory on a wheelchair, with a plaster splint along the left lower limb. She was in tachycardia up to 140 b/min, BP at 100/65 mmHg, liver was palpated 10 cm below the ribs; the spleen on umbilical level, had hard consistency and smooth surface. No signs of intracranial hypertension and meningoradicular irritation were detected.

**Figure 1:**
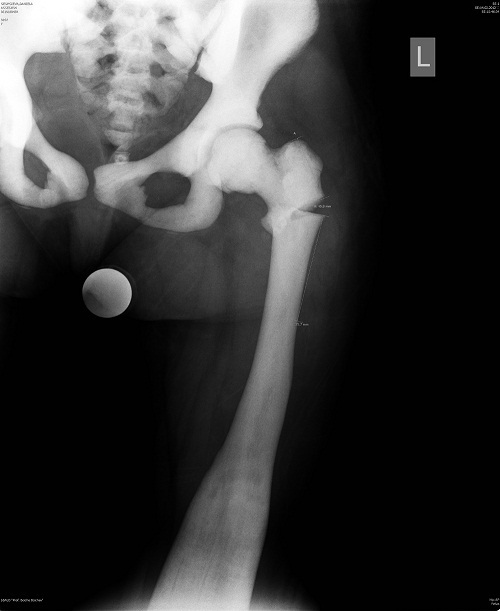
Diagnostic röntgenography

Ambulation was impossible. The left lower limb was fixed in an abduction and external rotation contracture. Each attempt for a passive movement in the left hip joint was extremely painful. AP and lateral X-rays of the left femur demonstrated intertrochanteric fracture with a slight varus deformity (Figure 1). The distal femur showed typical for the disease metaphyseal defect in a funnel form of an Erlenmeyer flask. Skeletal X-rays demonstrated typical diffuse sclerosis without differentiation between cortical and spongious bone, affecting skull, pelvis, spine, thorax and upper limb bones.

The preoperative hematologic investigation showed severe anemia with Hb 62 g/l, Htc 0.21, platelets 33.10 on 9/l, fibrinogen 6,2 g/l., without any other hematologic impairment. We performed preoperative substitution therapy with erythrocyte concentrate, plasma and platelets concentrate in sterile reanimation conditions. Operative treatment was performed on 20th of February 2012 with Hb 105 g/l, Htc 0,32, platelets 73 .10 on 9 /l.

**Figure 2:**
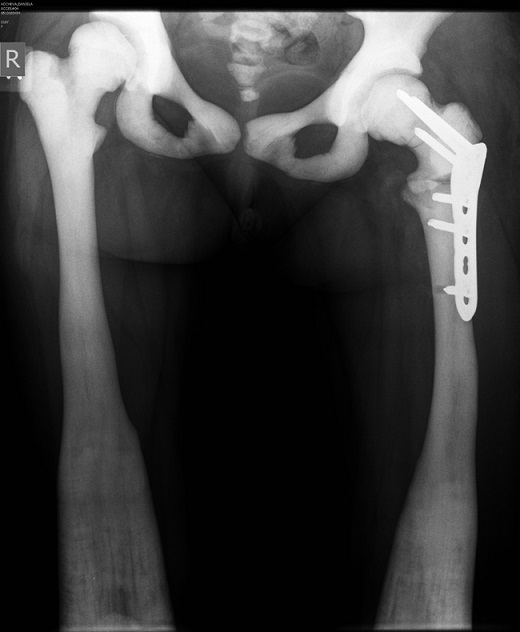
Postoperative roentgenographs – ORIF with 120° LCP Pediatric hip plate

Based on AO principles, we decided to perform surgical open reduction of the femoral fracture and fixation with a stable metal osteosynthesis. We used the technique with 120° LCP (Locking Compression Plate) Pediatric hip 5 mm. As we expected, the technical application of the synthesis was difficult due to bone hardness. We used a drill motor with a low speed and high torque, also brand new original steel drill bits. These bits were frequently replaced during the formation of the proximal three holes. Drill holes were inserted for a short distance with constant saline irrigation. Making of each hole took approximately 20 minutes. Perforation of the diaphysis for placement of the distal screws of the plate was not so difficult as was in the femoral neck because we used different, especially prepared for the case, diamond drill bits. These diamond drill bits were too short for the femoral neck length and could fit only for the diaphyseal holes. Duration of operation was 2 hours and 25 minutes with intraoperative blood loss of 270 ml. During proximal screws application appeared additional fissures in the femoral neck. They were detected on postoperative X-rays. These fissures did not compromise the stability of synthesis and the following bone consolidation (Figure 2). The early postoperative period was uneventful and standard antibiotic prophylactic was performed. Kinesitherapy with active movements was started on the first postoperative day. The child was gradually verticalized during the first postoperative week. Education in guided walking was started with a stable walker according to a special physiotherapy program, adapted for blind patients. Treatment continued with substitution therapy for the accompanying aplastic anemia by paediatric hematologist. A new physiotherapy course was performed for education in crutch walking after the third postoperative month. After the sixth month the child walks independently outside home only with a cane.

**Figure 3:**
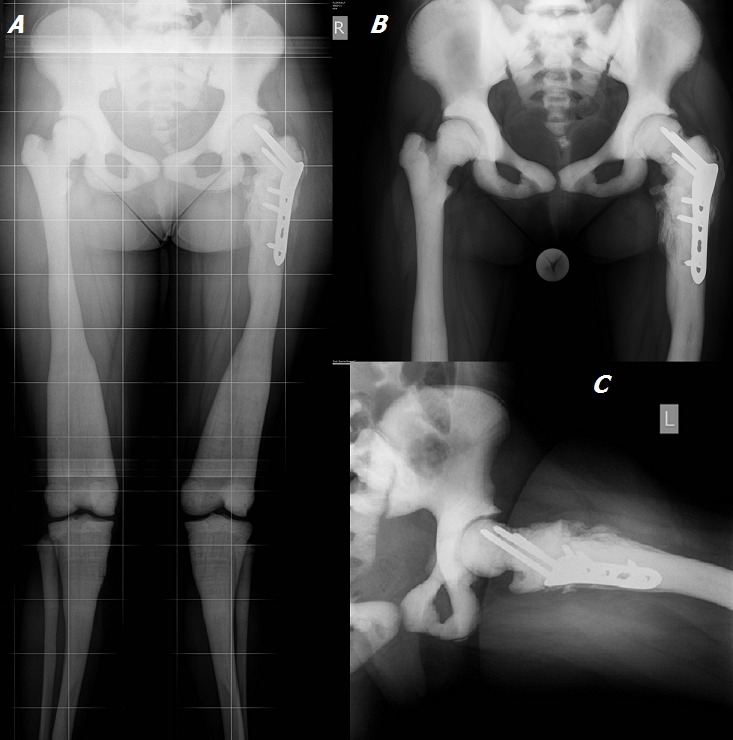
X-rays on 1 year post-operatively. Fracture consolidation, femoral neck-shaft angle of 132°, no lower limb length discrepancy

**Figure 4:**
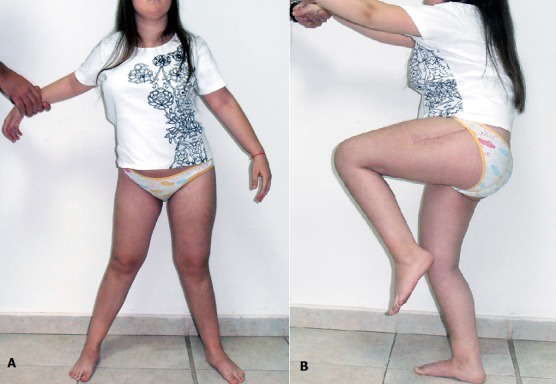
Hip range of active movements on 1 year postoperatively

On the X-rays in March 2013 (1 year postoperatively) we see consolidated fracture with a normal femoral neck-shaft angle. Lower leg length discrepancy is below 0.5 cm (Figure 3). Range of motion in the operated hip joint is: active flexion — 90°, extension — 5°, abduction — 30° (Figure 4). The patient is painless and her gait substantially improved. The child returned to her specialized for blind school for the new school year.

## Discussion

With a typical X-ray, the diagnosis of osteopetrosis is not difficult. Phenotype clarification of the disease type requires genetic counseling. In our case this wasn't done because of the lack of such possibility in Bulgaria. Clinical data alone in our case is not enough for type differentiation. On one side there were symptoms of osteopetrosis maligna — dolichocephalic configuration of the head, amaurosis, early hepatosplenomegaly and hematologic impairments, but on the other side, symptoms of osteopetrosis tarda were present — lack of early fractures, with the first fracture occurrence on 13 years of age, anamnesis of mandible osteomyelitis.

In the absence of genetic investigations, we assume that our case is most probably autosomal-dominant type II marble bone disease. The present fracture of the proximal femur in the described case is typical for the disease. This site is the most common localization of pathologic fractures in osteopetrosis [3]. Relatively rarer are the fractures of tibia, upper limb, cervical spine and spondylolyses. Usually the fracture line is transverse. Normal fracture callus develops, but healing is delayed due to faulty remodeling and maturation of the abundant newly formed bone. The newly formed callus envelopes like a bulb the old bone [4].

In children and adolescents, closed reduction and immobilization lead to successfull healing of mainly fractures of the upper extremity and tibia. The conservative treatment in the proximal femur is unreliable [5]. Pseudoarthrosis risk is high. Surgical treatment of these fractures is preferable. It requires thorough preoperative preparations because of high anaesthesiologic risk due to accompanying anemia and quite often lack of adequate control over the disease course.

The surgical intervention alone is difficult to perform due to the britle osteosclerotic bone during its technical preparation [6–8]. While drilling, the flutes of the drill bit are frequently filled with dense accumulated bone swarf, which reduces its effectiveness [8]. A large amount of heat is produced due to excessive friction and the risk of drill bit breakage is realistic. This demands usage of special drill motors and steel or diamond drill bits [9]. Frequent exchange of drill bits, cleaning of their flutes from the filling hard bone, constant saline cooling during drilling, are obligatory. All these difficulties were encountered intraoperatively also in our case. The demand for mechanical stability of the internal implants, used in marble bone disease, is higher than usual because of the longer stay of these implants over the slowly healing osteopetrotic bone; LCP pediatric hip meets absolutely this criteria. Applying this implant does not need bone deperiosting and does not impair bone circulation. The stable osteosynthesis allows early weightbearing. Another technical difficulty in osteopetrosis is preventing the implants to slip out of the extremely sclerotic bone. This demands usage of screws with fine threading such as the screws of the LCP pediatric hip.

Early postoperative physiotherapy in children with osteopetrosis is also a problem. It is due to the high incidence of visual impairments. In our case this problem was easily overcome due to the stability of the used of osteosynthesis. Physiotherapy was started immediately after the operation and was performed according to a program for blind children.

A possible complication in the early postoperative period is the development of osteomyelitis due to lack of intramedullary blood supply [4]. Another threat is the progression of the already present aplastic anaemia due to higher blood loss related to a longer intraoperative time. This necessitates regular check-up of haematologic indices and substitution therapy when indicated. All these complications were avoided in our case due to perfect interdisciplinary collaboration in therapy.

## Conclusion

Operative treatment of proximal femoral fractures in children with osteopetrosis is difficult, but not impossible. We assume that stable osteosynthesis after careful open reduction is obligatory. LCP pediatric hip is exactly such synthesis that guarantees successful treatment.
